# Self-Reported Pain and Symptoms in Community Dwelling Older Adults: Results from the Study of Aging II

**DOI:** 10.1093/geroni/igab046.3235

**Published:** 2021-12-17

**Authors:** Kristen Allen-Watts, Deanna Rumble, Cynthia Brown, Richard Kennedy

**Affiliations:** 1 University of Alabama at Birmingham, Birmingham, Alabama, United States; 2 Louisiana State University Health Sciences Center - New Orleans, New Orleans, Louisiana, United States

## Abstract

Pain is a common concern for community-dwelling older adults. There are a range of symptoms that may occur with pain that can be recurring and severe, which are associated with decreased quality of life. This study aims to characterize overall symptom load by utilizing the Brief Symptom Scale in community dwelling older adults who experience mild to severe pain. Data were extracted from the UAB Study of Aging II, a prospective, population-based study of mobility among community-dwelling older adults 75 years and older. Self-reported pain in the past 4 weeks and symptoms (e.g., pain, tired, nausea, depression, anxiety, shortness of breath) were included. The SPSS version 27.0 statistical package was used for analysis. Sixty-six percent were Non-Hispanic White, 58% were female, 40% lived in housing designed especially for the disabled, 49% were widowed, and 30% had a High School degree or GED. The mean age was 81 years (standard deviation 4.8). Of interest, over one third of the sample (38.1%) experienced moderate to severe pain, upper back pain was the most common area where pain occurred and feeling tired was the most common symptom. As the aging population continues to increase, so will the prevalence rates for pain. Findings suggests older adults with pain have multiple concomitant symptoms. Because the elderly represents a fragile and large group of the population, it is important to pay close attention to these symptoms.

